# Modulation of Energy Metabolism and Epigenetic Landscape in Rainbow Trout Fry by a Parental Low Protein/High Carbohydrate Diet

**DOI:** 10.3390/biology10070585

**Published:** 2021-06-25

**Authors:** Thérèse Callet, Hongyan Li, Pascale Coste, Stéphane Glise, Cécile Heraud, Patrick Maunas, Yvan Mercier, Nicolas Turonnet, Chloé Zunzunegui, Stéphane Panserat, Valérie Bolliet, Lucie Marandel

**Affiliations:** 1 INRAE, Université de Pau et des Pays de L’Adour, E2S UPPA, NUMEA, 64310 Saint-Pée-sur-Nivelle, France; therese.callet@inrae.fr (T.C.); lihy@ihb.ac.cn (H.L.); cecile.heraud@inrae.fr (C.H.); patrick.maunas@inrae.fr (P.M.); yvan.mercier@inrae.fr (Y.M.); nicolas.turonnet@inrae.fr (N.T.); czunzunegui97@orange.fr (C.Z.); stephane.panserat@inra.fr (S.P.); 2 State Key Laboratory of Freshwater Ecology and Biotechnology, Institute of Hydrobiology, Chinese Academy of Sciences, Wuhan 430072, China; 3 University of Chinese Academy of Sciences, Beijing 100190, China; 4 INRAE, Université de Pau et des Pays de L’Adour, E2S UPPA, ECOBIOP, 64310 Saint-Pée-sur-Nivelle, France; pascale.coste-heinrich@inrae.fr (P.C.); stephane.glise@inrae.fr (S.G.); valerie.bolliet@inrae.fr (V.B.)

**Keywords:** nutritional programming, teleost fish, offspring, broodstock, mitochondria

## Abstract

**Simple Summary:**

While the effects of parental diets on their progeny have been highly described in mammals, such studies are lacking in fish. To explore such a question in a high trophic level teleost fish, two-year old male and female rainbow trout were fed either a control diet (0% carbohydrate and 63.89% protein) or a high-carbohydrate diet (35% carbohydrate and 42.96% protein), for a complete reproductive cycle for females and for a period of 5 months for males. Neither the maternal nor the paternal high-carbohydrate diet alone had induced significant effects on their progeny. Nevertheless, when both parents were fed the high-carbohydrate diet, the energy metabolism and mitochondrial dynamics of their progeny were altered. Moreover, the epigenetic landscape was also highly affected. Even though, offspring growth was only slightly affected at the early stage of life; the effect of parental high-carbohydrate diet should be explored over the long term.

**Abstract:**

It is now recognized that parental diets could highly affect offspring metabolism and growth. Studies in fish are, however, lacking. In particular, the effect of a parental diet high in carbohydrate (HC) and low in protein (LP) on progeny has never been examined in higher trophic level teleost fish. Thus, two-year old male and female rainbow trout (*Oncorhynchus mykiss*) were fed either a control diet (0% carbohydrate and 63.89% protein) or a diet containing 35% carbohydrate and 42.96% protein (HC/LP) for a complete reproductive cycle for females and over a 5-month period for males. Cross-fertilizations were then carried out. To evaluate the effect of the parental diet on their offspring, different phenotypic and metabolic traits were recorded for offspring before their first feeding and again three weeks later. When considering the paternal and maternal HC/LP nutrition independently, fry phenotypes and transcriptomes were only slightly affected. The combination of the maternal and paternal HC/LP diets altered the energy metabolism and mitochondrial dynamics of their progeny, demonstrating the existence of a synergistic effect. The global DNA methylation of whole fry was also highly affected by the HC/LP parental diet, indicating that it could be one of the fundamental mechanisms responsible for the effects of nutritional programming.

## 1. Introduction

Exposure to environmental cues during phases with high plasticity (typically early life) can induce long-lasting changes in the morphology, physiology, and metabolism of an individual. This concept, referred to as programming, has been widely studied in various species and particularly for mammals with regard to the development of chronic diseases (developmental origins of health and disease (DOHaD)) [1]. Of particular interest is the nutritional programming, i.e., the consequences of nutritional stimulus that occur during the prenatal period [2], such as high carbohydrate (HC)/low protein (LP) parental diets [3,4,5]. Surprisingly, the numerous studies conducted on the effect of parental HC/LP diets in different species report strikingly common features [6,7]. For instance, it has been demonstrated that the offspring of parents fed HC/LP diets exhibit low birth weight [8]; metabolic disorders, such as impaired carbohydrate and lipid metabolisms [9]; and impaired cardiometabolic health [3,10].

Despite the increasing number of studies on nutritional programming, the mechanisms behind the imprinting of programming events and their subsequent effects on generations are not yet fully understood [11]. In mammals, several factors that can mediate the consequences of parental diet on the physiology of their offspring have been identified. The quality and quantity of nutrients provided via the mother’s diet are known to affect the phenotype of their offspring in the long term by directly affecting tissue development, which can lead to developmental alterations and defects in organ function [7]. Modifications of epigenetic marks are also a plausible mechanism to explain the effect of programming due to their susceptibility to environmental factors, their reversibility, and their role in the regulation of gene expression [2,12]. Dietary factors could alter different epigenetic marks, such as DNA methylation, either directly by affecting the availability of methyl donors or indirectly by affecting the availability of cofactors needed for enzymes involved in methylation processes [13]. Hence, HC/LP diets have been shown to induce either hypo- or hyper-methylation of DNA at specific sites [2]. Finally, increasing evidence also points to mitochondria as a key actor in nutritional programming. Disruption of mitochondrial activity due to nutritional events could alter the epigenome because of the role of mitochondria in producing important cofactors required for enzymes involved in epigenetic modifications [14].

In teleost fish, although only few studies have been conducted, the concept of programming has also been demonstrated in different species [15], and a particular focus has been given to aquaculture species [16]. Several studies have focused on different stimuli given during the early feeding phase and their long term consequences [17,18,19,20,21]. The consequences of stimuli applied earlier in development i.e., during gametogenesis, have also been tested. As such, the effects of a decrease in dietary methionine [22], complete plant-based diets [23], and selenium content [24] have been studied in rainbow trout. Nevertheless, the effects of parental HC/LP diets on their progeny have never been investigated in species with higher trophic levels. This question, however, is of importance as, for aquaculture sustainability, the diets of these species are evolving rapidly in farming practices and could trend towards an increase in the carbohydrate/protein ratio. Protein-rich fishmeal, traditionally used to formulate the diets of aquaculture species with a high protein requirement [25], could be replaced by plant-derived carbohydrates in broodstock diet [26,27]. It is thus essential to evaluate the consequences of a parental HC/LP diet on progeny in species with higher trophic levels.

To address this question, two-year old male and female trout were fed either a control diet formulated with no carbohydrate and a high protein content (NC diet, 0% carbohydrate and 63.89% protein) or a diet containing 35% carbohydrate but with a lower protein content (HC/LP, 35% carbohydrate and 42.96% protein) for an entire reproductive cycle for females and for a period of 5 months for males. Cross-fertilizations were carried out in order to obtain four groups of fry. Different phenotypic traits (mass, length, and morphometric analyses) were recorded before their first feeding. The effects on metabolism were investigated by measuring the energetic status of fry and by comparing their transcriptomes. The global DNA methylation was also assessed.

## 2. Material and Methods

### 2.1. Experimental Design

**Figure 1:**
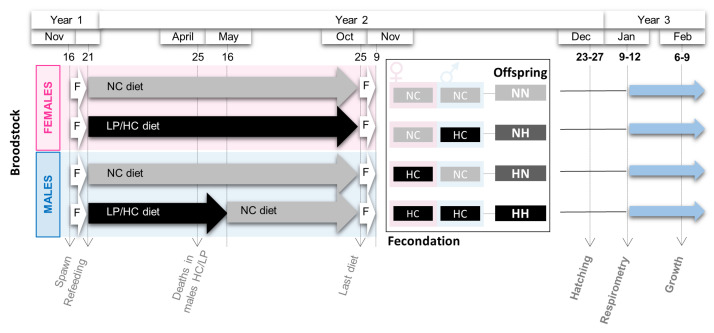
Experimental design. Broodstock females and males were fasted (F) after spawn from 16 Nov (November) to 21 Dec (December). Then, they were fed for one year either the NC diet (no-carbohydrate) or the HC/LP (high carbohydrate/low protein) diet (see details in Material and Methods Section). From the 25 of October (Oct) to the 9 of November, broodstock fish were fasted again. Cross-fertilizations were then carried out to obtained four groups of offspring: NN, NH, HN, and HH. Respirometry analyses and samplings were performed after hatching and just before the first feeding. Offspring were then fed for 3 weeks, and a second sampling was performed.

Two-year old male and female rainbow trout (*Oncorhynchus mykiss*) were distributed into two 8 m${}^{3}$ tanks at 8 ${}^{\circ }$C (November—year 1) and fed since the resumption of feeding in the experimental INRAE facilities of Lees-Athas (Figure 1). Broodstock were fed either a control diet containing 63.89% protein and 0% carbohydrate (NC) or a diet containing 42.96% protein and 35.30% carbohydrate (HC/LP). Fishmeal was used as the protein source, fish oil was included as the lipid sources, and carbohydrates were provided by gelatinized starch (Table S1). These two diets were prepared in our own facilities (INRAE, Donzacq, France) as extruded pellets (BC45 BisVis Clextral, France). The feeding experiment lasted until the next reproduction for females, i.e., 10 months (October-year 2). Regarding males, a *Saprolegnia* sp. infection occurred in late April (year 2) in the tank with males fed the HC/LP diet, and it triggered some deaths. To keep a sufficient amount of fish alive for reproduction, the males were re-fed the NC diet (details in Callet et al. [27]).

During the spawning period (November—year 2), spawns from 3 NC females and 2 HC/LP females were cross-fertilized with milts from 4 males from each experimental condition (NC and HC/LP diets). Thus, offspring from 4 different conditions were obtained: NN, fry from both males and females fed the control diet; HN, fry from only females fed the HC/LP diet and males fed the control NC diet; NH, fry from only males fed the HC/LP diet and females fed the NC diet; and HH, fry from both parents fed the HC/LP diet (Figure 1).

Eggs hatched in December (year 2), from 44 to 48 days post-fertilization (dpf) (HH: from 44 to 46 dpf; HN: from 44 to 47 dpf; NH: from 45 to 48 dpf; and NN: from 46 to 48 dpf). Fry were divided in two distinct batches. To investigate the specific effects of the parental HC/LP on metabolism, some yolk-sac fry were transferred before complete resorption to an experimental INRAE facility (Saint-Pée-sur-Nivelle, France), and they were kept at 10 ${}^{\circ }$C. Experiments were run before the first feeding between 61 and 64 dpf (description below). To monitor growth, some yolk-sac fry were transferred to another experimental INRAE facility (Donzacq, France), where they were kept at 17 ${}^{\circ }$C. At complete resorption, fry were fed a commercial diet (T3-P Omega, Skretting, France). After 3 weeks (Feb-year 3), fry were weighed and individually photographed from both sides.

### 2.2. Metabolic Rate Assays

At 61 dpf, over a 4-day period (January—year 3), 16 yolk-sac fry were randomly selected (n = 4 per condition) to measure their metabolic rates (MR). Individuals were kept for 24 h at 10 ${}^{\circ }$C (±0.1 ${}^{\circ }$C) under dim light (7 lx) for acclimatization and then placed in respirometry chambers of an intermittent flow respirometer (Loligo Systems, Viborg, Denmark) as previously described by Régnier [28]. Oxygen consumption as a proxy for MR was measured over an 18 h period at 10 ${}^{\circ }$C. Six hours were removed from the data, as this period is considered the acclimation period. The standard metabolic rate (SMR) was estimated by calculating the oxygen consumption during the last three hours of measurement (3 slopes). For the routine metabolism (RMR), oxygen consumption was measured over 12 h after the acclimatization period (12 slopes). SMR and RMR are expressed in mm${}^{3}$·O${}_{2}$·g${}^{-1}$ of wet mass. After MR measurement, individuals were anaesthetized in a benzocaine bath at 30 mg·L${}^{-1}$, weighed (BW), and then killed in a benzocaine bath at 60 mg·L${}^{-1}$. A photo of each yolk-sac fry was taken from both sides. The samples were then individually frozen in liquid nitrogen and stored at −80 ${}^{\circ }$C for further analyses.

### 2.3. Fry Body Morphologies

Body and yolk-sac measurements were performed for each fry (left and right side) by two different people using the software ImageJ (https://imagej.nih.gov/ij/index.html, accessed on 1 March 2020). To characterize fry body morphologies (before complete resorption and after 3 weeks of feeding), the fork length (L), the body depth (D) the head length (HL), the pre-orbital length (POL), and the whole-body area (WBA) were measured (Figure S1). Eye morphologies were also assessed by measuring the eye diameter (ED) of each fry. The relative head length (HLr), relative pre-orbital length (POLr), and relative eye diameter (EDr) were calculated by dividing HL, POL, and ED by fry length. Fulton’s condition factor, K, was calculated as follows: K = 100 × BW/L${}^{3}$. To describe fry yolk-sac morphologies (i.e., yolk-sac utilization), the yolk-sac length (YSL), height (YSH), and area (YSA) were measured. Then, the relative yolk-sac area (YSAr), height (YSHr), and length (YSLr) were calculated by dividing YSA, YSH, and YSL by WBA, D, and L, respectively. The yolk-sac volume (YSV) was estimated as follows: YSV = (π/6) × YSL × YSH${}^{2}$ [29].

### 2.4. RNA and DNA Extraction

Whole yolk-sac fry tissues were individually homogenized in ice using an Ultra Turrax homogeniser (T25 basic IKA-WERKE) at the speed setting of 4 for 10 s until they were homogeneous. Total RNA, DNA, and protein were extracted from whole yolk-sac fry (n = 16 per condition) using the QIAGEN AllPrep DNA/RNA/Prot Preparation Kit according to the manufacturer’s recommendations (Qiagen). The concentration of extracted RNA was analyzed using a spectrophotometer (Nanodrop ND1000, LabTech) by measuring absorbance at 260 nm. The quality of RNAs was checked with a Bioanalyzer (Agilent Technologies, Kista, Sweden), and 12 samples for each condition were selected for further analyses according to the RIN (RNA integrity number).

### 2.5. Determination of Fry Sex

The extracted DNA was used to assess the sex of the fry (n = 12 per condition), as previously described in Yano et al. [30]. Briefly, PCR was performed to amplify the master sex-determining gene (sdY gene). Then, 1 μL of DNA was mixed with 1.25 μL of each primer at 10 μm (forward: CCCAGCACTGTTTTCTTGTCTCA; reverse: CTGTTGAAGAGCATCACAGGGTC), 1 of μL dNTP mixture (Ozyme, 4 × 10 mM), and 5 μL of 5xPCR Buffer (Promega) with 0.125 μL of Taq DNA polymerase (Promega, 5 U/μL) in a total volume of 25 μL. Thermal cycling consisted of denaturation for 20 s at 94 ${}^{\circ }$C followed by 35 cycles of 94 ${}^{\circ }$C for 20 s, 59 ${}^{\circ }$C for 20 s, and 72 ${}^{\circ }$C for 20 s, with a final extension of 5 min at 72 ${}^{\circ }$C. PCR products were electrophoresed on a 2% agarose gel in a 1.5× TAE buffer and stained with SYBRSafe to reveal the presence or absence of sdY.

### 2.6. Global DNA Methylation

According to manufacturer recommendations, 1.5 μg of DNA (measured by Nanodrop) was digested with 0.5 μL of RNase cocktail (Ambion, Austin, TX, USA) containing 500 U/mL of RNaseA and 20,000 U/mL of RNase T1 to avoid further nucleoside contamination. Digestion was conducted for 30 min at 37 ${}^{\circ }$C. Nucleosides were obtained through a single step hydrolysis process using DNA Degradase Plus (Zymo Research, Orange, CA, USA) following the manufacturer’s protocol. In brief, the reaction mix consisted of 1.5 μg DNA sample, 2.5 μL 10× DNA Degradase Plus reaction buffer, 1 μL DNA Degradase Plus (5 U/μL), and H_2_O ultrapure up to a total of volume of 25 μL. The reaction mixes were incubated at 37 ${}^{\circ }$C for 2 h followed by heat inactivation at 70 ${}^{\circ }$C for 20 min. Samples were ultracentrifuged using 0.5 mL of Amicon Ultra at 3 kDa units (Merck Millipore, Billericia, MA, USA) according to the manufacturer’s instructions.

HPLC-UV analysis was based on the protocol described in Kovatsi et al. [31] with major modifications. The system consisted of an Alliance 2695 separation module (Waters, Milford, MA, USA), a 2487 dual Absorbance Detector (Waters, Milford, MA, USA), and a column oven. Chromatographic separation was performed on a Luna C8 (3 μm, 100 × 3 mm) (Phenomenex, Torrance, CA, USA) column. The mobile phase was composed of the following: solvent A: 10 mM potassium phosphate buffer, pH = 5.9 ± 0.1; solvent B: 100% methanol. Linear gradient elution was employed as follows: 0, 100% A; 0–8 min, 90% A; 8–8.5 min, 73% A; 8.5–13.5 min, 65% A. The flow was set at 0.5 mL/min. The temperature of the column oven was 25${}^{\circ }$C. The wavelength of UV detection was 277 nm.

Identification and quantification of nucleosides were conducted with external standards purchased from Berry and Associates (Dexter, MI, USA). The monitored nucleosides were 2′-deoxycytidine (dC), 5-methyl-2′-deoxycytidine (5-mdC), 5-hydroxymethyl-2′-deoxycytidine (5-hmdC), 5-formyl-2′-deoxycytidine (5-fdC), and 5-carboethoxy-2′- deoxycytidine (5-cadC). The in vivo global level of 5-mC, 5-hmC, 5-fC, and 5-caC was calculated as the percentage of each individual’s molar quantity divided by the total molar quantity of all the detected cytosine forms. Taking 5-mC as an example, the percentage of 5-mC was calculated using the following equation: 5-mC% = 100 × Q5-mC/(QC + Q5-mC + Q5-hmC + Q5-fC + Q5-caC), where QC, Q5-mC, Q5-hmC, Q5-fC, and Q5-caC are the molar quantities of 5mC, 5hmC, 5-fC, and 5-caC, respectively.

### 2.7. Microarrays, cDNA Labelling and Hybridization

Transcriptome profiles of yolk-sac fry were analyzed with microarray technology (n = 12 per condition). Microarray analyses were performed on an RBT-specific Agilent-based microarray platform with 8 × 60 K probes per slide. Then, 150 ng of total RNA was first amplified by a reverse transcription using a polyDT T7 primer (denaturation step: 10 min at 65 ${}^{\circ }$C; reaction step: 2 h at 40 ${}^{\circ }$C; inactivation step: 5 min at 70 ${}^{\circ }$C). The obtained cRNA were labelled with Cy3-dye (2 h at 40 ${}^{\circ }$C). Excess dye was removed using a RNeasy kit (Qiagen). The level of dye incorporation was evaluated using a spectrophotometer (Nanodrop ND1000, LabTech; yield > 0.825 µg cRNA and specific activity > 6 pmol of Cy3 per µg of cRNA). A total of 600 ng of Cy3-cRNA was then fragmented with a specific buffer (30 min at 60 ${}^{\circ }$C). Cy3-cRNAs were then hybridized on a sub-array (17 h at 65 ${}^{\circ }$C in a microarray hybridization oven (Agilent). Slides were washed and scanned (Agilent DNA Microarray Scanner, Agilent Technologies, Massy, France) using the standard parameters for a gene expression 8 × 60 K oligoarray (3 µm and 20 bits). Data were then collected with the Agilent Feature Extraction software (10.7.1.1) and are available in GEO (accession number: GSE171310).

### 2.8. qPCR

Expressions of some genes selected in the transcriptomic analyses and genes from different pathways of interest were assessed by qPCR. Assays were carried out according to MIQE (Minimum Information for Publication of Quantitative Real-Time PCR Experiments) standards [32]. For each sample (n = 12 per condition), 1 μg of total RNA per condition was reverse transcribed to cDNA with SuperScript III RNase H reverse transcriptase (Invitrogen, Carlsbad, CA, USA) using random dT Primers. qPCRs were performed using the Roche Lightcycler 480 system (Roche Diagnostics, Neuilly-sur-Seine, France). Then, 2 μL of diluted cDNA (2 μL cDNA diluted in 150 μL of water) was mixed with 3 μL of LightCycler 480 SYBR Green I Master Mix and diluted to obtain a final volume of 6 μL. Forward and reverse primers were used at a final concentration of 400 nM. Thermal cycling was initiated with an incubation at 95 ${}^{\circ }$C for 10 min. Forty five steps of PCR were performed, each one consisting of heating at 95 ${}^{\circ }$C for 15 s for denaturing and at 60 ${}^{\circ }$C for 10 s for annealing as well as a third extension step at 72 ${}^{\circ }$C for 15 s. Melting curves were systematically monitored (with a gradient of 0.5 ${}^{\circ }$C/10 s from 55 ${}^{\circ }$C to 94 ${}^{\circ }$C) to confirm the specificity of the amplification reaction. Each PCR assay was run with replicate (duplicate of reverse transcription and PCR amplification) and negative controls included. PCR efficiency was measured by the slope of a standard curve using serial dilutions of cDNA (5 dilutions of a pool of all conditions from D20 to D380 in triplicate). Primer sequences and PCR efficiency values are presented in Table S2.

### 2.9. Statistical Analyses

All the statistical analyses were performed using R Software (version 3.2.5) [33]. Linear mixed-effects models were used to analyze the different parameters measured using the packages “lme4” from R software with a significance threshold set at *p*-value = 0.05. The best model was then selected using the Akaike Information Criterion (AIC). Concerning fry phenotypes, the effects of the maternal HC/LP diet and paternal HC/LP diet as well as the interaction between these two variables were investigated via the zootechnical parameters measured and the yolk-sac measurement. The sampling day and fry identification (except for BW and K) were treated as random effects. Then, the correlations between yolk-sac measurements and body measurements were estimated using a Pearson test (*cut-off p* = 0.01). When a significant correlation between two traits was present, ANCOVA analyses were used to test if the relationship between those traits was affected by the parental HC/LP diet.

To investigate the potential effect of the parental HC/LP diet on fry metabolism, the maternal nutritional history, the paternal nutritional history, and the interaction between these variables were investigated using the SMR and RMR. Then, the correlations between the SMR, RMR, yolk-sac, and body measurements were also estimated (Pearson test). When a significant correlation between two traits was present, ANCOVA analyses were used to test if the relationship between them was affected by the parental HC/LP diet.

Data from the microarray analysis were transformed with a logarithmic transformation, scale normalized, and analyzed using the Limma package [34]. In order to identify the differentially expressed genes resulting from the maternal, paternal, and parental HC/LP diet, transcriptomes of HN, NH, and HH fry were successively compared with the transcriptomes of NN fry. For these three comparisons, Limma *t*-tests were performed with a correction for multiple tests (*cut-off p* = 0.05 after a Benjamini–Hochberg correction), taking into account the sex of the fry and the time of the sampling. To characterize the change induced by the parental HC/LP diet, the gene ontologies of the probes that were annotated were collected from the DAVID (Database for Annotation, Visualization and Integrated Discovery bioinformatics resource, version 6.7) [35].

The relative expressions of genes assessed by qPCR were calculated by a mathematical method based on the real-time PCR efficiencies (Table S2) using a geometric mean from three reference genes (*eef1a*, *actb* and *18S*) for normalization [36]. The relative mRNA levels obtained were then transformed with a logarithmic transformation. To investigate the potential effect of the parental HC/LP diet on these mRNA levels, the effects of the sex of the fry, the maternal nutritional history, the paternal nutritional history, and the interaction between these last two variables were investigated using linear mixed-effects models. In situations where a significant interaction was present, a Tukey post hoc test was carried out. The day of sampling was treated as a random effect. As previously described, AIC was used to select the best fitted model.

## 3. Results

### 3.1. Phenotypes

**Figure 2:**
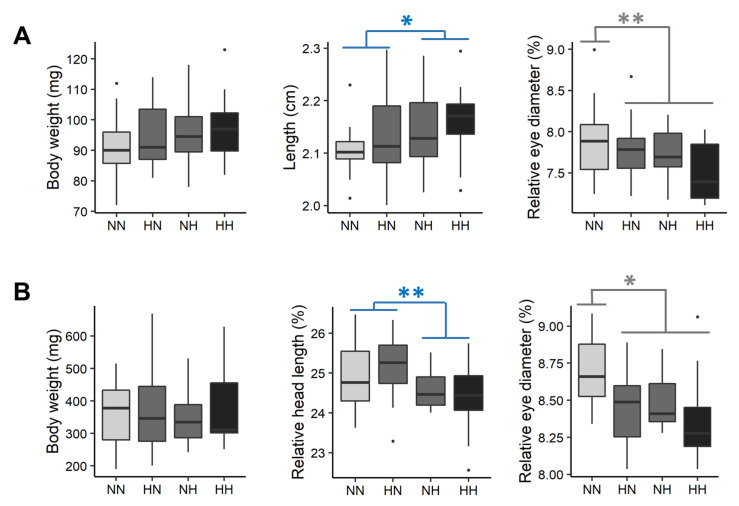
Effect of the parental HC/LP on fry body mass, length, relative head length, and relative eye diameter (**A**) before first feeding and (**B**) after 3 weeks of feeding. The effect of the paternal nutritional history is depicted in blue and the effect of both the maternal and the paternal nutritional history in grey (”**” means *p*-value < 0.01 and ”*” means *p*-value < 0.05).

Fry phenotypes, including fry body mass, body, head, and yolk-sac morphologies, were recorded before their first feeding and three weeks later. Between 61 and 64 dpf (before first feeding), the whole yolk-sac fry weighed 94.91 ± 10.49 mg (Figure 2A). Except for the fry total length and the relative eye diameter (Figure 2A), no significant differences were detected among fry body morphologies (i.e., for BW, K, D, WBA, HL, HLr, POLr, and ED, see Table S3 for statistical details). NH and HH fry had a significantly higher total length than that of NN and HN fry (+1.5%, $\chi^{2}$ = 4.0, df = 1, *p* = 0.046). The relative eye diameters of HN, NH, and HH fry were, respectively, 2.55, 2.32, and 3.78% lower than those for NN fry. Regarding yolk-sac morphologies (YSH, YSHr, YSL, YSLr, VYS, YSA, and YSAr), no significant differences were detected among offspring (Table S3).

After 3 weeks of feeding, fry weighed 362.41 ± 106.75 mg (Figure 2B). No significant differences were detected in fry body morphologies (i.e., for BW, K, L and D). However, the head proportions of NH and HH fry were on average 2.44% lower than those of HN and NN fry ($\chi^{2}$ = 10.7, df = 1, *p* = 0.001). Moreover, the eye proportions of HN, NH, and HH fry were 1.62, 1.93 and 4.94% lower, respectively, than those of NN fry ($\chi^{2}$ = 4.9, df = 1, *p* = 0.03) (Figure 2B).

**Figure 3:**
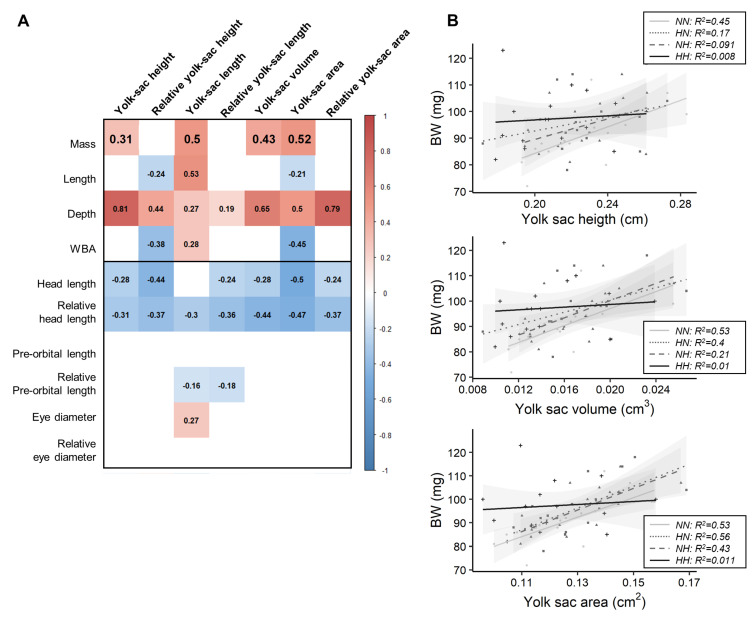
Relationships between growth and yolk-sac measurements. (**A**) Matrix of correlation between traits. Correlation were investigated using Pearson tests (*cut-off p* = 0.01), and each value is indicated inside the matrix, while significant positive correlations are represented in red significant negative ones in blue. (**B**) Relationships between fry mass and yolk-sac height, volume, and area according to parental dietary history.

Before the first feeding, phenotypical traits related to the body (BW, K, L, D, and WBA) and head (HL, HLr, POLr, and ED) morphologies were correlated with different yolk-sac traits (Figure 3A). All but three of these relationships remained unaffected by the parental nutrition. The relationships linking BW and yolk-sac length, volume, and area were significantly different for HH fry in comparison to those for NN fry (Figure 3B). While NN fry body mass was positively correlated with YSH, VYS and YSA, such correlations did not exist for HH fry.

### 3.2. Metabolic Rate

**Figure 4:**
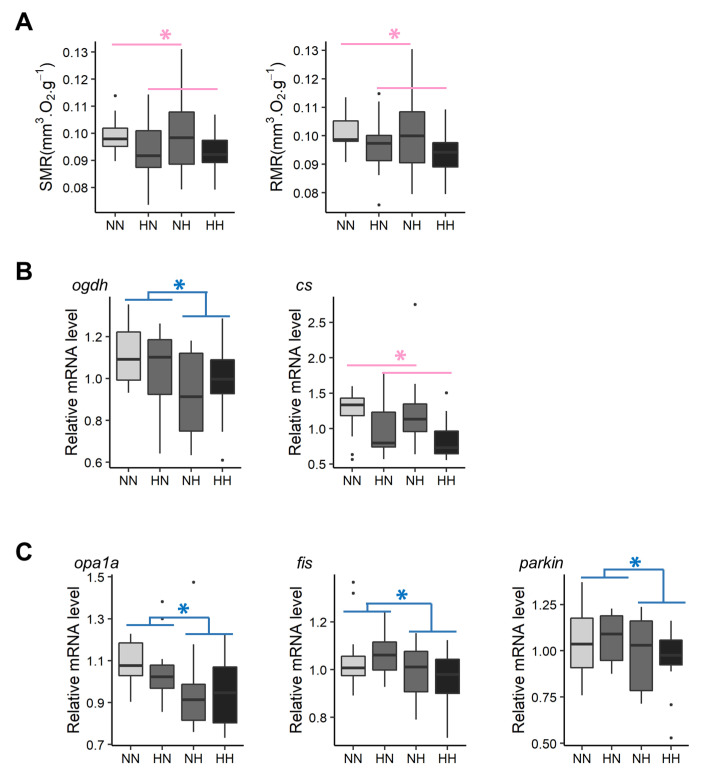
Effect of parental HC/LP on fry metabolism and, in particular, (**A**) on fry metabolic rates, (**B**) relative mRNA levels of genes related to energy production, and (**C**) relative mRNA levels of genes involved in mitochondrial dynamics. The effect of maternal nutritional history is depicted in red and the effect of paternal nutritional history in blue (”*” means *p*-value < 0.05).

Fry metabolic rates were measured between 61 and 64 dpf (Figure 4A). The HH and HN fry had on average a 6.45% and a 5.48% lower SMR and RMR, respectively, than those of NH and NN fry ($\chi^{2}$ = 5.4, df = 1, *p* = 0.02 and $\chi^{2}$ = 4.3, df = 1, *p* = 0.04, respectively). Moreover, neither SMR nor RMR were significantly correlated with yolk-sac measurements (*p* > 0.05).

### 3.3. Global DNA Methylation

**Figure 5:**
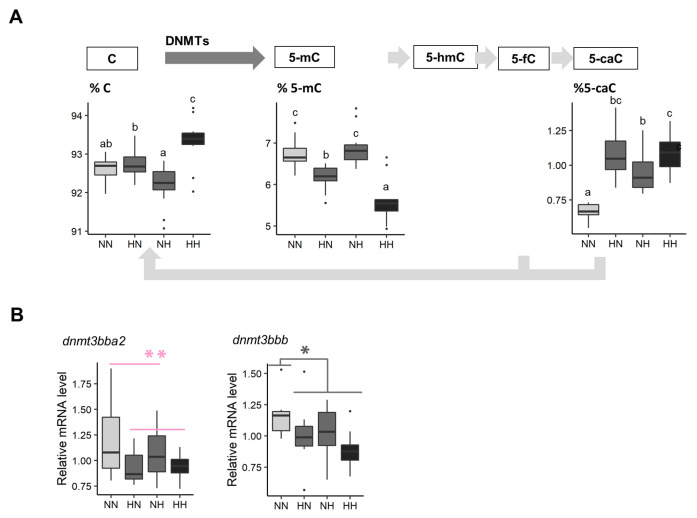
Effect of parental HC/LP on fry epigenetic landscape. (**A**) Proportion of intermediates of DNA methylation in their offspring and (**B**) relative mRNA levels of two homologs coding for Dnmt3, an enzyme involved in the maintenance of DNA methylation patterns. The effect of the maternal nutritional history is depicted in red and the effect of both the maternal and the paternal nutritional history in grey (”*” means *p*-value < 0.05). Different letters indicate significant differences between groups, which were investigated with a Tukey post hoc test, in the case of a significant interaction between the paternal and the maternal nutritional history.

The five intermediates of DNA methylation were measured in whole fry between 61 and 64 dpf (Figure 5A). The C proportion was significantly decreased in HN and HH in comparison to the C proportion observed in NN fry ($\chi^{2}$ = 12.2, df = 1, *p* < 0.001). The 5-mC proportion was 8.3% and 16.8% lower in HN and HH fry, respectively, in comparison to that observed in NN fry ($\chi^{2}$ = 9.3, df = 1, *p* = 0.002). The 5-hmC and 5-fC were not detected by HPLC-UV in our samples. Finally, the 5-caC proportion significantly increased in HN, NH, and HH fry in comparison to that observed in NN fry ($\chi^{2}$ = 11.9, df = 1, *p* < 0.001).

### 3.4. Transcriptomes

Between HN and NN fry, no significant difference in expressions of probes was found, while 15 probes were differentially expressed between NH and HH fry, and only the ankyrin repeat domain 16 had a fold change (FC) > 1.5 (Table S4). In total, 308 probes were differentially expressed between HH and NN fry, of which 8 had a FC > 1.5 (Table S4). Among these probes, 7.1% were not annotated. Regarding the annotated probes, the most represented pathways were those involved in metabolic pathways (18.2%), regulation of transcription (17.9%), immunity (8.8%), development (including genes coding for crystallin), and the signaling pathway.

Pathways related to metabolism included genes coding for glucose transporters and genes involved in glycolysis and amino acid metabolism, energy production, and mitochondrial dynamism. Hence, these pathways were selected to be further analyzed by qPCR, thereby allowing for the validation of microarray data (Figure S2). Thus, HN and HH fry had, on average, 13.6% and 14.6% higher *hk2* and *pfkla* mRNA levels, respectively, when compared with those of NH and NN fry. On average, NH and HH fry had significantly lower *glut1ba* (−14.8%), *glut1bb* (−9.4%), *pfkmaa* (−19.5%), *pfkmab* (−26.8%), *pfkmba* (−39.3%), and *pfkmbb* (−21.9%) mRNA levels than those of HN and NN fry. HN fry had significantly higher *ldhaa* mRNA levels than those of the fry from the three other conditions. Results regarding genes coding for glucose transporters and the 6-phosphofructokinase are in accordance with results obtained by the transcriptomes analyses. In regard to amino acid catabolism, NH and HH had, on average, significantly lower *hibadh* mRNA levels than those of NN and HN fry (−18.6%). In contrast, no differences in the levels of mRNA for genes coding for the glutamine synthetase and glutaminase were detected among all conditions, which is in contrast with results obtained from the transcriptomic analyses. Regarding energy production, mRNA levels from one of the three homologs coding for the citrate synthase were decreased in HN and HH fry in comparison with those of NN and NH (−26.1%). Moreover, mRNA levels for one of the three homologs coding for 2-oxoglutarate dehydrogenase decreased by −11.1% in NH and HH fry when compared with the levels recorded for NN and HN (Figure 4B). Together, these results are in accordance with the transcriptomic analyses. Interestingly, the comparison of transcriptomes also revealed some effects of the parental HC/LP diet on mitochondrial dynamism (*bnip3* and *mfn2*). qPCR results did not confirm the results obtained for mitofusin 2 (*mfn2*). NH fry tended to have a lower *bnip3a* mRNA level than that of NN fry. Moreover, NH and HH fry had, on average, significantly lower *opa1a* (−10.4%), *fis* (−8.4%), and *parkin* (−9.1%) mRNA levels than those of NN and HN fry (Figure 4C).

Finally, the most significant result according to the transcriptomic analyses (Table S4) concerns a gene that codes for the DNA methyltransferase 3 (log2 fold change = −2.02). Among the 8 homologs known to code for this protein in trout, *dnmt3bba2* mRNA levels were, on average, significantly decreased in HN and HH fry in comparison to those of NN and NH (−15.9%). *dnmt3bbb* mRNA levels were also −15.3% lower in HN, NH, and HH fry in comparison to those of the control NN (Figure 5B). Finally, NH, HN, and HH fry also had −20.0% lower *crybbb* mRNA levels (gene coding for crystallin) than those of NN.

## 4. Discussion

In an effort to make aquaculture more sustainable, much focus has been placed on broodstock diets in which fishmeal has been replaced by terrestrial plants products, thus increasing their carbohydrate content [26,27]. However, parental HC diets are known to significantly impact both the phenotypes of offspring as well as metabolism in mammals [2]. The effects of such parental diets have yet to be explored in higher trophic level teleost fish. The present study sought to evaluate the impact of a maternal and paternal HC/LP diet individually in addition to the combination of the maternal and paternal HC/LP diet, namely, the parental HC/LP diet, on yolk-sac fry. During this stage, fry switch from an endogenous to exogenous feeding and thus undergo important metabolic changes, which could reveal some changes triggered by the programming led by parental nutrition.

### 4.1. Offspring Growth Was Unaffected by the Parental HC/LP Diet

First, several measurements were carried out to detect any adverse effects of the parental HC/LP diet on offspring phenotypes, especially with respect to growth.

Neither the maternal nor the paternal nutritional history affected offspring body mass. During this early stage, the yolk-sac was not yet fully resorbed, and the mean mass included not only the mass of the fry but also the mass of the remaining yolk sac. As such, some effect of the parental diet on growth could have be hidden if body mass had been considered alone. Body morphology parameters, such as total length, depth, and whole body area, needed to be used as a proxy for growth. Among the different morphological measurements that depict growth, a slight increase was recorded only for the total fry length in response to the paternal HC/LP diet. However, this effect was small and was not observed after 3 weeks of feeding. Overall, the parental HC/LP diet did not significantly affect offspring growth in rainbow trout. This result is of particular interest as it is in direct contrast with results typically found in mammals [3,37,38,39,40,41,42,43,44,45] and birds [46] in which both maternal and paternal LP diets are known to compromise offspring growth.

In fish, in contrast to mammals, all of the nutrients available for an individual’s development and growth before the first feeding are deposited in the egg, facilitating the study of programming. It has previously been demonstrated that female rainbow trout fed a challenging diet could maintain the quality of their eggs [26,47]. Such findings have been confirmed in the present experiment. Indeed, egg size, which reflects the quantity of nutrients available and is known to be correlated with the fry mass at first feeding in rainbow trout [48], was not significantly affected by the HC/LP diet [27]. Moreover, egg macronutrient content (glycogen, free glucose, lipid, and protein) was also maintained [27]. The unimpaired quality of eggs (nature of macronutrients and quantity provided) produced by females fed the HC/LP diet accounts for the fact that offspring growth was maintained. Furthermore, we also investigated the possible effect of the parental diet on the rate of yolk-sac resorption. The negative relationship between yolk-sac measurements, fry growth, and, in particular, head length, was expected, as the more the fry grows, the more its yolk-sac diminishes [49]. These relationships were not affected by the parental HC/LP nutrition. Thus, offspring could have used their reserves at a similar rate, regardless of their parental nutritional history, explaining why no differences in fry growth were detected at this early stage. Moreover, positive relationships were observed between fry mass–fry depth and yolk-sac measurements, which probably illustrates the fact that the biggest fry had the biggest yolk-sac, regardless of their resorption rate state. Interestingly, while there are positive relationships between BW and yolk-sac height, BW and volume, and BW and area for NN fry, such relationships did not exist for the HH fry. That is, while the heaviest NN fry had the biggest yolk-sac (area and volume), this was not the case for HH fry. A large variability in HH fry size along with differences in resorption rate amongst HH fry may explain this result. However, such variability in resorption rates was not related to a greater variability in metabolic rates (Figure 4) and should be further explored.

### 4.2. Eye Development Was Affected by the Parental HC/LP

Interestingly, while fry growth did not seem to be strongly affected by the parental nutritional history, both paternal and maternal HC/LP diets significantly decreased the relative eye diameter in fry. Interestingly, this effect persisted after 3 weeks of feeding. At both time intervals, a synergistic effect of the paternal and maternal diets appeared as the HH fry had the lowest relative eye diameter. In addition, an important number of genes differentially expressed between the HH and the control NN fry were linked to neurological development pathways. Among them, relative mRNA levels for several genes coding for crystallin, the protein responsible for the optical properties of the lens [50], were significantly decreased in HH fry (log2FC = −0.5). Interestingly, in rainbow trout, eye size is correlated with age and not fish size, as eye growth is known to be maintained during a period of restricted feeding, while somatic growth is typically decreased [51].

Despite the fact that the reduction of protein in the parental HC/LP diet did not impair offspring growth, eye development could have been affected. The effect of a maternal LP diet on hormonal signals should be explored. Hormonal signals have been identified as a potential mechanism that can explain the imprinting of maternal nutrition. Of particular interest is cortisol, which is deposited in eggs and is also known to affect fry and eye development [52,53].

### 4.3. Energy Metabolism Was Altered by the Parental HC/LP Diet

To assess any adverse effects of the parental HC/LP diet on yolk-sac fry, metabolic rates were measured and non a priori analyses were used to detect any pathways affected.

The paternal HC/LP diet did not impair metabolic rates in offspring, and their transcriptomes were not highly affected. These results were not awaited. It is now well recognized that sperm also carry information that could highly affect their offspring metabolism in the long term [9] via changes in sperm epigenetic status [54]. Male broodstock could have been selected by the important mortality triggered by the *Saprolegnia* sp. [27]. Following this event, a dietary shift occurred during the feeding trial, and, as a consequence, male broodstock only received the HC/LP diet for a period of 5 months. Both the potential selection of males and the restricted exposure to HP/LC diet could explain the limited effect observed for offspring phenotypes and metabolism.

In contrast, while the maternal HC/LP diet induced a shift in offspring metabolic rates, no differences were detected at the molecular level (i.e., transcripts) between the HN and NN fry.

Finally, a synergistic effect of paternal and maternal nutritional histories was observed for metabolism. HH fry had lower metabolic rates than those of the NN control. Moreover, this result is in accordance with results obtained at the molecular level. The comparison of fry transcriptomes revealed that the HH fry metabolisms were more affected than the control NN were. The combined effect of the paternal and maternal HC/LP diet led to dysregulation of energy metabolism at the transcriptomic level, as seen by the overall downregulation of the expression of genes coding for proteins involved in energy production (glycolysis and TCA) in HH fry in comparison to NN fry. Previous studies reported a similar outcome in the muscle of offspring born from mothers fed LP diets in murine species [8,55,56].

Mitochondria, the powerhouse of the cell, are dynamic organelles that can either fuse to create more elongated mitochondria or a mitochondrial network, or undergo fission, thus creating a fragmented mitochondrial network. In murine species, maternal nutrient insults, such as a high sugar diet or a LP diet, typically induce unbalanced mitochondrial fusion/fission, leading to mitochondrial dysfunction in offspring across different generations [57,58,59,60,61]. In addition to the previous results regarding bioenergetics, qPCR also revealed that relative mRNA levels in genes coding for Bnip3 (log2FC = −0.13 between NH and NN), Opa1 (log2FC = −0.18 between HH and NN), and Fis (log2FC = −0.14 between HH and NN), proteins that orchestrate either mitochondrial fusion or fission, were slightly affected by the parental HC/LP diet. Interestingly, even though there is a synergistic effect of paternal and maternal HC/LP diets, the effect is primarily due to the paternal HC/LP diet, suggesting that the imprinting of the programming event is more likely due to epigenetic modulation than due to the maternal transmission of defect mitochondria [61].

Gene expression can vary greatly from one tissue to another and can be regulated in the opposite direction. This is particularly true for genes involved in mitochondrial morphology and dynamics [62]. Moreover, the processes of mitochondrial fusion and fission are subjected to complex regulations through transcription and both post-transcription and post-translational regulation [62,63,64]. In the present study, due to the size of the fry at this early stage, analyses were conducted on whole fry. Despite being relative *parkin* mRNA levels, a gene coding for a protein involved in the mitophagy pathway, which can occur after mitochondrial fission, was also significantly reduced in NH and HH fry (log2FC = −0.14). It is impossible to confirm whether fission was promoted by the paternal HC/LP diet. Despite this limit, these preliminary results illustrated that a parental HC/LP diet could affect the balance between mitochondrial fusion and fission, which need to be tightly regulated to maintain an adequate mitochondrial function and, thus, energy metabolism in offspring.

Finally and more importantly, even though mitochondrial dynamics and energy metabolism were altered by both the maternal and the paternal HC/LP diets at the transcriptomic level and metabolic rates were disturbed, fry growth were not highly affected. Moreover, no correlation between metabolic rates and growth parameters (BW, length, depth, and WBA) was observed. Nevertheless, metabolic rates are known to be positively correlated with growth in fish (i.e., fish with higher SMR grew faster when fed *ad libitum*) [65]. This striking result suggests that the changes observed in metabolism were not strong enough to compromise fry growth. However, as eye development was affected by the parental HC/LP diet, it could be hypothesized that the parental HC/LP modified the allocation of fry energy.

### 4.4. The Parental HC/LP Diet Induced Global DNA Hypo-Methylation

The inheritance of epigenetic information is now recognized as the main mechanism underlying the effect of nutritional programming, and among them is the modification of DNA methylation [13]. Here, we demonstrated that the maternal HC/LP diet induced global DNA hypomethylation in offspring, with a synergistic effect of the paternal HC/LP diet. The effect of alteration of the DNA methylation landscape and the modification of relative mRNA levels of genes involved in the methylation/demethylation process by the maternal HC/LP diet have previously been demonstrated in murine species [2]. While the biological role of this intermediate of the DNA demethylation pathway still needs to be assessed [66], both the paternal and the maternal HC/LP diets induced a significant augmentation of 5-caC. This provides further evidence that the HC/LP diet could have impact on the epigenetic landscape.

Different hypotheses have been proposed to explain how a parental HC/LP diet could alter the epigenetic landscape in offspring. First, it was previously shown that maternal HC/LP diets could directly affect *dnmt1* and *dnmt3* expression by alteration of the 1-carbon metabolism [12]. These genes code for the enzymes responsible for the maintenance of DNA methylation patterns during cell replication (Dnmt1) and de novo addition of a methyl group (Dnmt3). Such outcomes have been described in the liver of the offspring in mammals [67,68]. Interestingly, in the present study, the maternal HC/LP diet also induced an alteration of the expression of two homologs coding for Dnmt3. More specifically, relative *dnmt3* mRNA levels were significantly reduced in HH and HN fry (log2FC = −0.32 and −0.38). The potential different functions of these two homologs are still currently unknown [69], but these results are in line with the reduced proportion of 5-mC in these two groups.

LP/HC diets could also alter the availability of methyl donors or the cofactors needed for enzymes involved in methylation/demethylation processes, in which mitochondria play a central role. Mitochondria, whose role is not only restricted to energy production, also produce important cofactors required for enzymes involved in epigenetic modifications, such as alpha-ketoglutarate, which mediates DNA demethylation processes catalyzed by the ten-eleven translocation enzyme. Thus, the reprogramming of the mitochondrial function in an altered nutrient environment could also strongly impact the epigenetic landscape [14]. For this reason, additional studies are needed to further investigate and describe the effect of the parental HC/LP diet on mitochondrial dynamics.

## 5. Conclusions

In rainbow trout, a teleost fish, we showed that a parental HC/LP diet affects offspring energy metabolism in the short term. However, growth was not compromised at this early stage in contrast to results typically observed in mammals. The carbohydrate/protein ratio could be further increased in the broostock diet, and plant-derived carbohydrates thus appear as an efficient substitute to fishmeal. However, it is necessary to test the effect of the parental diet in the long term, because the epigenetic landscape (global DNA methylation) was profoundly impacted, and some effects of the nutritional programming can only be revealed later in fish life, particularly during periods of stress. Finally, at both the molecular and phenotype levels, the effects triggered by the maternal HC/LP diet seemed to accumulate with the paternal effects, despite the fact that they were only fed for a period of 5 months. Thus, particular attention should be given to the synergistic effect existing between paternal and maternal nutritional histories.

## Data Availability

Data are available on the GEO website under the accession number: GSE171310.

## Supplementary Materials

The following are available at https://www.mdpi.com/article/10.3390/biology10070585/s1, Figure S1: Descriptions of the different body measurements. D: body depth; HL: head length; POL: the pre-orbital length; ED: eye diameter; YSL: yolk-sac length, and YSH: yolk-sac height. Figure S2: Log2 fold changes obtained with microarray (purple) and with qPCR (grey) analyses. Table S1: Diet composition and fatty acid profile (% total FA). Table S2: List of the primers used for qPCR analyses. Table S3: Results of the linear mixed-effects used to investigate the effect of the sex of the fry and the parental HC/LP diet on the phenotypical traits recorded, SMR/RMR, methylation pattern, and gene expression. For each trait, the chosen model (based on AIC) is indicated in bold. Table S4: Results of the transcriptomic analyses: list of probes differentially expressed between NH and HH and HH and HH fry in addition to their fold change (FC) and the corrected *p*-value obtained by Limma *t*-tests.
