# Supplementary material for: Modulation of Energy Metabolism and Epigenetic Landscape in Rainbow Trout Fry by a Parental Low Protein/High Carbohydrate Diet
Source: Biology (Basel). 2021 Jun 25;10(7):585. doi: 10.3390/biology10070585 (PMC8301017; doi:10.3390/biology10070585)
Supplement: Supplementary file 1 [file biology-10-00585-s001.zip › TableS1.pdf]

A.

	Diets	
	NC	HC
**Ingredients (%)**		
Fish meal[1]	77.77	45
Pregelatinized starch[2]	-	37
CPSP 90[3]	2.00	5
Soya meal[4]	12	-
Soy protein concentrate[5]	-	5
Fish Oil[6]	1.66	3.96
Cellulose[7]	2.53	-
Alginate[8]	2	2
Mineral Premix[9]	1	1
Vitamin Premix[10]	1	1
Carophyll pink[11]	0.04	0.04
**Proximate composition**		
Dry Matter (DM,%)	91.84	96.22
Crude Protein (%DM)	63.89	42.96
Crude Lipid (%DM)	8.90	9.2
Gross energy, kJ/g DM	20.69	20.38
Ash, % DM	15.93	9.71
Carbohydrates, % DM	<0.2	34.30

B.

	Diets	
	NC	HC
Saturated		
14:0	11.55	10.57
15:0	1.13	1.42
16:0	27.34	29.53
18:0	2.83	4.35
Total saturated	43.46	46.97
MUFA		
16:1	7.66	7.86
18:1	15.39	15.69
20:1	4.60	2.06
Total MUFA	30.52	26.98
PUFA n-6		
18:2 n-6	3.57	2.14
20:4 n-6	0.62	1.22
Total PUFA n-6	4.19	3.36
PUFA n-3		
18:3 n-3	1.14	1.18
18:4 n-3	2.09	1.61
20:4 n-3	0.15	0.00
20:5 n-3	5.87	6.39
22:6 n-3	5.84	7.22
Total PUFA n-3	15.09	16.39
LC-PUFA n-3	11.86	13.60
Sat/PUFA	1.99	2.10
n3/n6	3.60	4.88

Table S1. Diet composition and fatty acid profile (% total FA). NC, no-carbohydrate diet; HC, high-carbohydrate diet, DM : dry matter, MUFA: monosaturated fatty acids, PUFA: polyunsaturated fatty acids LC-PUFA: long chain polyunsaturated fatty acids

[1]Sopropeche, Boulogne-sur-Mer, France. [2]Gelatinized corn starch; Roquette, Lestrem, France. [3]Sopropêche. [4]Sudouest aliment.[5]Legouessant.[6]Fish oil; Sopropeche, Boulogne-sur-Mer, France.[7]Upae.[8]Louis François.[9]Supplied the following (/kg diet): calcium carbonate (40% Ca) 2.15 g, magnesium oxide (60% Mg) 1.24 g, ferric citrate 0.2 g, potassium iodide (75% I) 0.4 mg, zinc sulphate (36% Zn) 0.4 g, copper sulphate (25% Cu) 0.3 g, manganese sulphate (33% Mn) 0.3 g, dibasic calcium phosphate (20% Ca, 18% P) 5 g, cobalt sulphate 2 mg, sodium selenite (30% Se) 3 mg, potassium chloride 0.9 g, sodium chloride 0.4 g. Louis François, Marne-la-Vallée, France.[10]Supplied the following (/kg diet): DL-a tocopherol acetate 60 IU, sodium menadione bisulphate 5 mg, retinyl acetate 15,000 IU, DLcholecalciferol 3,000 IU, thiamin 15 mg, riboflavin 30 mg, pyridoxine 15 mg, vit. B12 0.05 mg, nicotinic acid 175 mg, folic acid 500 mg, inositol 1,000 mg, biotin 2.5 mg, calcium panthotenate 50 mg, choline chloride 2000 mg.[11]Astaxanthine, DSM
