# Supplementary figures and images for: Modulation of Energy Metabolism and Epigenetic Landscape in Rainbow Trout Fry by a Parental Low Protein/High Carbohydrate Diet
Source: Biology (Basel). 2021 Jun 25;10(7):585. doi: 10.3390/biology10070585 (PMC8301017; doi:10.3390/biology10070585)

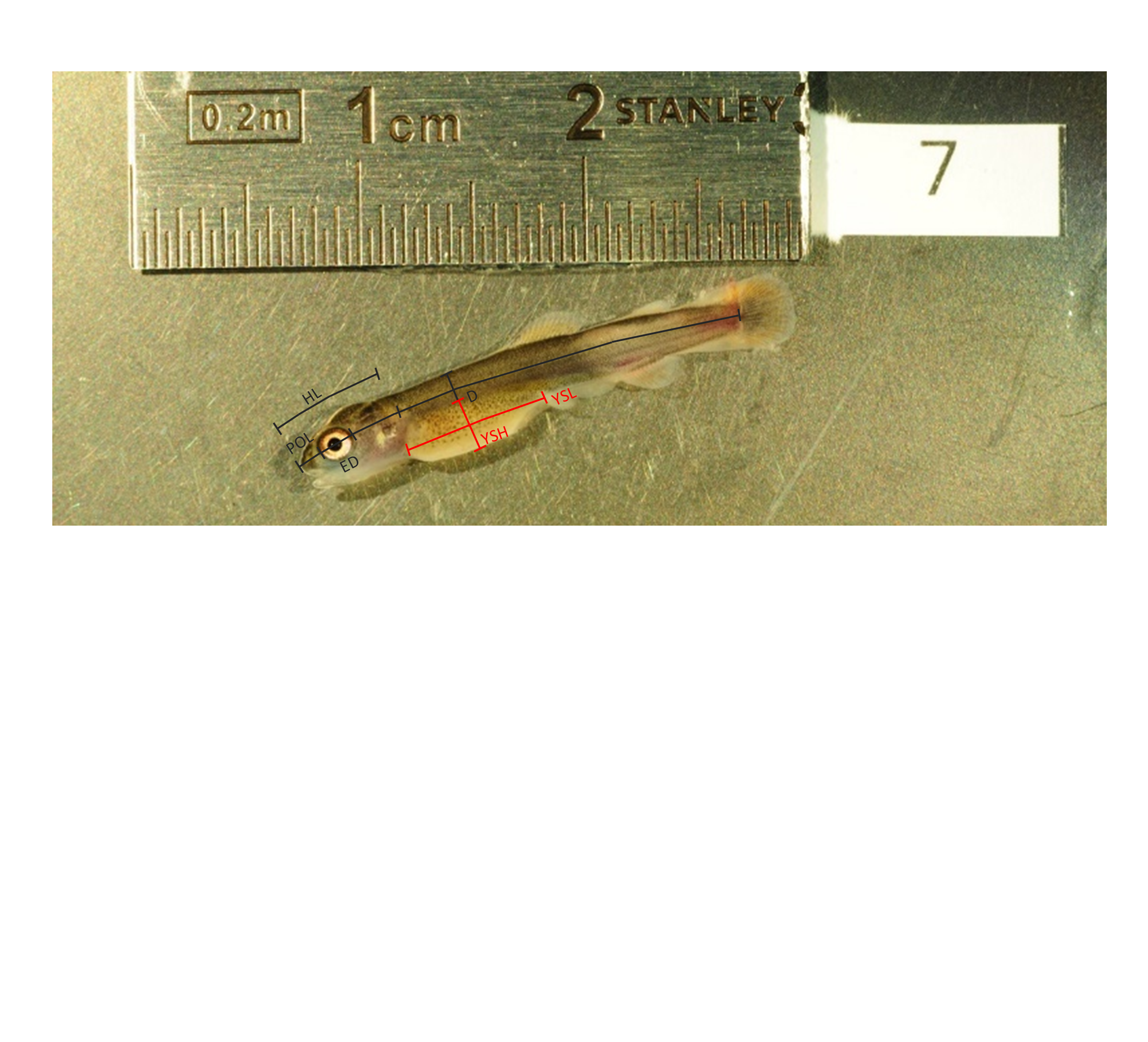

Supplement: Supplementary file 1 [file biology-10-00585-s001.zip › Figure S1.png]

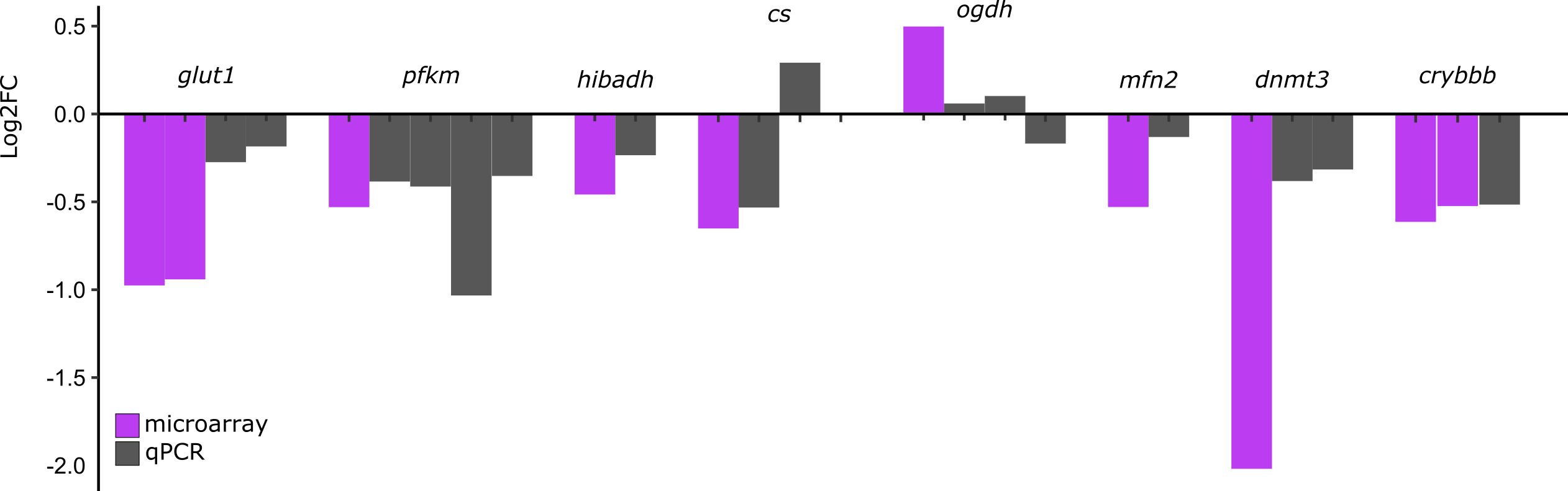

Supplement: Supplementary file 1 [file biology-10-00585-s001.zip › FigureS2.png]
